# Prevalence and Characteristics of the Maxillary Sinus Septa in a Saudi Arabian Sub-population: A Retrospective Cone Beam Computed Tomography (CBCT)-Based Study

**DOI:** 10.7759/cureus.47605

**Published:** 2023-10-24

**Authors:** Muath S Alassaf, Albraa Alolayan, Esam Almuzaini, Abdulmajid A Masoudi, Khalid Alturki, Ahmed K Alsaeedi, Bakr M Sedqi, Shadia A Elsayed

**Affiliations:** 1 Orthodontics and Dentofacial Orthopedics, Taibah University, Madina, SAU; 2 Oral and Maxillofacial Surgery, Taibah University, Madina, SAU; 3 Dental Education, Taibah University, Madina, SAU; 4 Dentistry, College of Dentistry, Taibah University, Madina, SAU; 5 Periodontics, Ohud Dental Center, Madina, SAU; 6 Oral and Maxillofacial Surgery, Al-Azhar University, Cairo, EGY

**Keywords:** maxillary implant, sinus morphology, implant dentistry, ridge augmentation, dental implant, sinus lift, sinus septa, maxillary sinus septa

## Abstract

Objectives: This study aims to investigate and determine the prevalence, location, and morphological differences of the maxillary sinus septa using cone beam computed tomography (CBCT) images in the Madina region and to discuss its clinical implications during sinus surgery.

Methods: This study was a retrospective cross-sectional radiographic analysis of 808 CBCT scans from patients who attended Taibah University's College of Dentistry between December 2018 and June 2023. Age, gender, number of septa present per side, location (anterior, middle, posterior), morphology (complete or partial), and type (primary or secondary) were among the study variables. For data statistics, correlation analysis was utilized.

Results: Eight hundred eight CBCT scans were included with a mean age of 34.7±15.08. Septal bone was absent in 60.64% of the sinuses examined. Single septa were more common than multiple septa (p=0.009). Bilateral septal presence and the left septal bone were more common than the right, but none of these distributions were statistically significant. Males showed more sinus septa compared to females, but the difference was not statistically significant (p=0.420). Single septa were more prevalent in people under the age of 25.

Conclusion: More than 60% of the Madina population had patent sinus cavities with no septal processes, but males with single septal processes predominated.

## Introduction

Maxillary sinus septa (MSS) divide the sinus into many compartments, irregular cortical bone projections from the sinus floor [1]. According to Underwood's theory, he first noticed it as it emerged during the tooth eruption phases. Krennmair also classified septa into primary and secondary septa. The main septa form during the early stages of the maxilla's development, whereas the secondary septa appear due to tooth loss, which will cause the maxillary sinus to become pneumatized [2].

In order to ensure adequate implant insertion, the edentulous maxillary posterior region frequently needs augmentation operations, including sinus floor elevation, using either the osteotome or lateral window approach [3]. Schneiderian membrane perforation, which is shown to be the most prevalent complication to occur with 7 to 56% of these procedures, is one of many consequences, including oro-antral fistula, sinus inflammation, and other issues [4].

Any implant insertion should be preceded by a comprehensive treatment plan, especially in regions with nearby essential structures. cone beam computed tomography (CBCT) has provided various inspections and information for the past 20 years [5]. Because of CBCT's great resolution, surgeons can see the bony component of the maxillary sinus, which allows them to identify and treat any septa during the surgery [6].

Forty-one percent of the 9631 patients in 42 trials with 42 studies exhibited sinus septa [7]. In Saudi Arabia, the sinus septa were the subject of various investigations, revealing them to be substantial, with percentages ranging between 37.64% and 46% [8]. However, a study from Riyadh found a different result, showing that only 5% of the CBCTs under examination exhibited septa [9,10].

Knowing the common types and anatomical information is essential to improve our knowledge of the population databases because sinus septum morphology may affect sinus lift surgery and may impact cases of foreign body removal or membrane elevation procedures. Therefore, this study aims to investigate and determine the prevalence, location, and morphological differences of the maxillary sinus septa using CBCT images in the Madina region and to discuss its clinical implications during sinus surgery.

## Materials and methods

Study design 

A retrospective cross-sectional radiographic analysis was performed on CBCT scans obtained from patients who attended Taibah University's radiology department between December 2018 and June 2023. All patients aged 18 and above with no history of sinus surgery and pathology that interferes with septa clarity were included. CBCTs with poor quality such as blurring and segmented CBCTs were excluded. An 808 randomly selected CBCT scans from Taibah University's College of Dentistry were examined by one of the investigators between December 2018 and June 2023 to look for any maxillary septa and to count, locate, and analyze the patterns of maxillary sinus septa. The sample size was calculated using guidelines that closely matched the Saudi population: 80% power, a 95% confidence interval (CI), and a 0.05 margin of error.

Study variables

Patient demographic data were collected from patient's medical records on the college R4 system (R4 Clinical and Practice Management Software database archives, (CS Health, Inc. Rochester, NY, USA) and included the following: age, gender, presence of septum, uni-bi maxillary septum, location (anterior, middle, posterior), morphology (full or partial), and type (primary or secondary).

Data collection technique

**Figure 1:**
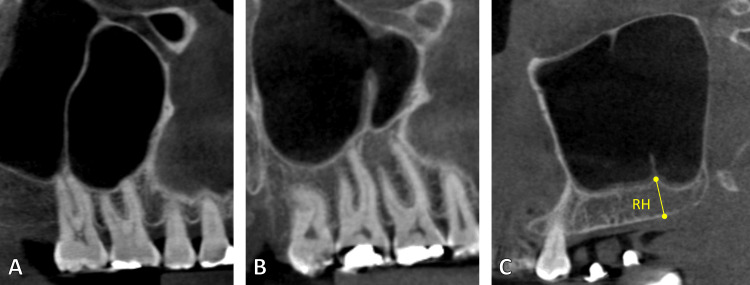
The extension of the maxillary sinus septum can be complete (A) or partial (B). The ridge height (RH) is measured with a line parallel to the present septum

**Figure 2:**
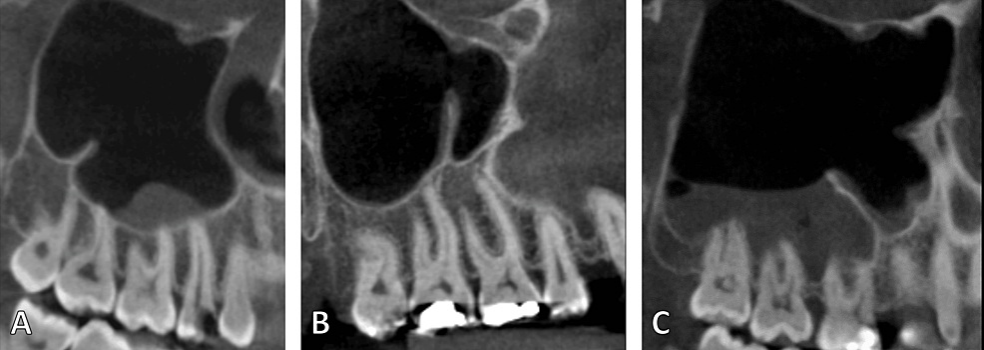
The maxillary sinus septum showed on a sagittal cut of CBCT showing posterior (A), middle (B), and anterior (C) locations of the septum according to its relation with the teeth CBCT: cone beam computed tomography

The three anatomical sites of the sinus septa were anterior (bicuspid area), middle (between the first and second molars), and posterior (distal to the second molar). Septa connected to an edentulous ridge were classified as secondary, whereas septa connected to a maxillary tooth were classified as primary. The septa are regarded as complete vertically or horizontally, depending on how far they extend toward the neighboring wall. On the other hand, the partial septum is absent from the opposing side (Figures 1, 2). The axial, sagittal, and coronal planes were used to assess the presence of maxillary sinus septa. A line was drawn to measure the distance between the septa's lowest and highest points. After being trained and calibrated, two examiners conducted all of the measures. The same measurements for each CBCT were collected, and then the average was calculated.

Ethical consideration

Each radiograph received an anonymous code, and all the data was recorded in an Excel file (Microsoft, Redmond, WA, USA) using a security password. Taibah University's ethical committee authorized the study (TUCDREC110923/MSAlassaf). All patient information was kept anonymous. As the study used existing radiographic records and no individual identifiable information was presented, a waiver from consent was approved by the ethics committee.

Data statistical analysis

We used Statistical Package for Social Science 16 (SPSS, version 16, Chicago, IL, USA) to analyze the data from an Excel sheet. The sample characteristics were reported using descriptive analysis. Continuous variables were presented as (M+SD) mean with standard deviations if the data coincides with normality distribution (Kolmogorov-Smirnov p>0.05). Analysis of the qualitative variables were as frequency and percentages. ‎‏The significance level was set at p-value ≤0.05.

## Results

The study included 808 CBCT scans of patients with a mean age of 34.7±15.08. Septa number, position, angulation, and correlation with demographic patient data of age and gender were all study variables. Septal bone was not present in 60.64% of the investigated sinuses. There were 94 septa in the right sinus (Table 1). Over 14% of people had bilateral septal presence. The left side was more prevalent than the right, but none of these distributions were statistically significant (Table 2).

**Table 1:** The prevalence of maxillary sinus septa in the Madina, Saudi Arabia population according to gender * Cramer’s V and Phi value is 0.191 with approximate significance 0.009 and (df=3)

Variable	Female (n=160)	Male (n=648)	Total	P-value
Presence and side of septa (n=808)				.420
Not Present	92 (11.39)	398 (49.26)	490 (60.64)	
Bilateral	25 (3.10)	91 (11.24)	116 (14.36)	
Rt Only	24 (2.97)	70 (8.66)	94 (11.63)	
Lt Only	19 (2.35)	89 (11.01)	108 (13.37)	
Total number of septa per individual (n=318)				
One	38 (11.95)	145 (45.60)	183 (57.55)	.009*
Two	23 (7.23)	87 (27.36)	110 (34.59)	
Three	2 (0.63)	16 (5.03)	18 (5.66)	
Four	5 (1.57)	2 (0.63)	7 (2.20)	

**Table 2:** The prevalence of maxillary sinus septa in the Madina, Saudi Arabia population according to age groups * Cramer’s V and Phi value is (.094) and (.164) with approximate significance 0.010 and (df=9) ** Cramer’s V and Phi value is (.100) and (.172) with approximate significance 0.020 and (df=12)

Variable	Below 25 (n=239)	25 to 35 (n=194)	36 to 45 (n=201)	Above 45 (n=174)	Total	P-value
Presence and side of septa (n=808)						.010*
Not Present	167 (20.69)	114 (14.09)	111 (13.74)	98 (12.13)	490 (60.64)	
Bilateral	17 (2.10)	36 (4.45)	37 (4.58)	26 (3.22)	116 (14.36)	
Rt Only	24 (2.97)	22 (2.72)	27 (3.34)	21 (2.60)	94 (11.63)	
Lt Only	31 (3.84)	22 (2.72)	26 (3.22)	29 (3.59)	108 (13.37)	
Total number of septa per individual (n=318)						
One	52 (16.35)	38 (11.95)	47 (14.78)	46 (14.47)	183 (57.55)	.020**
Two	18 (5.66)	34 (10.69)	34 (10.69)	24 (7.55)	110 (34.59)	
Three	1 (0.31)	6 (1.89)	6 (1.89)	5 (1.57)	18 (5.66)	
Four	1 (0.31)	2 (0.63)	3 (0.94)	1 (0.31)	7 (2.20)	

Males showed more sinus septa than females, but the difference was not statistically significant (p=0.420), and single septa were more common than multiple septa (p=0.009). The middle position was more common than the anterior and posterior positions, and most septa were only partially present (Table 3).

**Table 3:** Distribution of septa in terms of location, morphology, type, and measurements based on the side (n=487)

Variable	Left (n=254)	Right (n=233)	Total Septa (n=487)
Location			
Anterior	44 (9.03)	46 (9.44)	90 (18.49)
Middle	133 (27.32)	127 (26.12)	260 (53.44)
Posterior	77 (15.82)	60 (12.32)	137 (28.13)
Morphology			
Partial	251 (52.51)	225 (47.07)	476 (99.58)
Complete	3 (0.63)	8 (1.67)	11 (2.30)
Type			
Primary	173 (36.15)	168 (35.15)	341 (71.30)
Secondary	81 (16.94)	65 (13.56)	146 (30.54)
Measurements, mean (SD)			
Septal height	5.31 (±3.7)	5.91 (±5.5)	5.59 (±4.1)
Ridge height	11.34 (±4.6)	11.58 (±4.8)	11.46 (±4.7)

**Figure 3:**
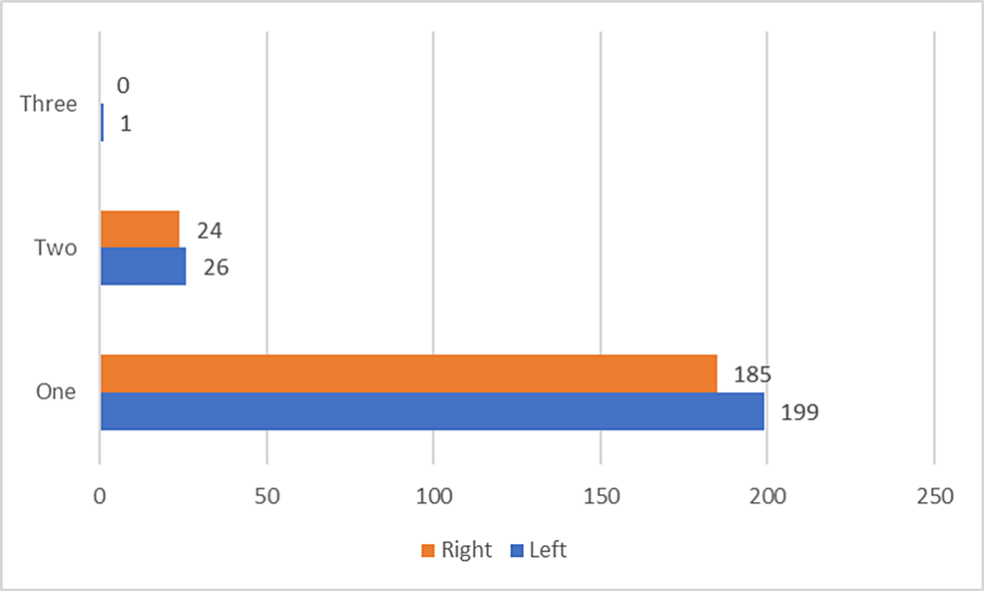
Distribution of cases based on the number of maxillary sinus septa per side

Single septa were more common in adults under 25, while double septa were more common in people over that age (Figure 3). Correlation between right and left septal height was 0.375 with significance level (p<0.000). In addition, positive correlation between right and left ridge height was also noted (0.385) with statistical level (p<0.000).

## Discussion

The maxillary sinus is divided into several compartments by bony structures called maxillary sinus septa, a common anatomical normal variation [9]. Individuals can have different levels of maxillary sinus septa prevalence and distribution. Using CBCT images in the Madina region, the current study aimed to analyze and determine the septa's prevalence, position, and morphological variations and address its clinical significance following sinus surgery. The current investigation found that 39.4% of the sample had sinus septa. One or both maxillary sinuses of the same person may have septa, while others may not. There were no statistically significant gender differences, although other research demonstrated that gender statistically impacts septal frequency [10]. Other investigations with similar findings to those in the current study demonstrated a male predominance in the septal presence [11,12].

These septa are categorized into two forms based on their location: complete septum and incomplete septum. A complete septum splits the maxillary sinus into two separate smaller sinus compartments. A partial septum is a bony ridge or crest providing a smaller partition within the maxillary sinus but not entirely dividing it. Infraorbital recess septum, lateral recess septum, or median recess septum. They can range in thickness from small and delicate to solid and bony.

Different imaging methods have been used by radiologists, otolaryngologists, and oral and maxillofacial surgeons [13]. Typically, computed tomography (CT) scans or CBCT, which we used in the current investigation, are used to detect the presence of maxillary sinus septa [14]. To appropriately diagnose and treat patients, radiologists, and dental professionals should correctly detect and evaluate the septa. Treatment planning involves placing dental implants or endoscopic sinus surgery to modify the procedure and reduce the risk of complications [15,16].

Maxillary sinus septa can complicate surgical sinus procedures, interfere with the drainage and ventilation of the sinus, and have other clinical implications. Therefore, while planning operations or assessing sinus-related difficulties like sinus lifts for dental implant insertion or endoscopic sinus surgery, radiologists and dental professionals frequently consider the presence and type of maxillary sinus septa [17]. Oral surgeons must carefully identify these septa to reduce complications during surgery, such as perforation of the sinus Schnederian lining or harm to nearby tissues like the infraorbital nerve or the maxillary artery. Furthermore, the sinus septum may raise the risk of chronic sinusitis and recurrent infections, which may refer pain and infection to the dental alveolus and vice versa [18].

Depending on the population studied and the diagnostic techniques chosen, studies have indicated various prevalence rates for maxillary sinus septa, ranging from about 20% to 45% or even more. These prevalence percentages can vary depending on gender, age, and ethnicity. It's crucial to remember that these rates are approximations and may differ dramatically between patient populations and geographic areas. In the present study, a large percentage of the population had no septa in 60.64% of the cases examined. This outcome was consistent with those of others who identified septa in about 37-46% of the individuals they studied [6,19,20].

Other investigations that contradict the current findings [6] found that right-side septa were more prevalent [6,21]. However, Altayar et al. observed that the left side was more dominant than the right side, consistent with our findings [22]. Other researchers, such as Paknahad et al., attempted to compare the frequency of sinus septa in cleft and non-cleft individuals and observed no difference in the prevalence of MSS between patients with cleft lip and palate (CLP) and non-CLP individuals [23]. Dental professionals participating in sinus-related operations and treatments should know the anatomy of the maxillary sinus septa. It aids in surgical planning, lowers the potential of complications, and ensures the best possible patient outcomes [24,25].

Limitations and recommendations 

A worth-mentioning limitation of this study is the predominantly male composition of the sample, which may not fully represent the prevalence and characteristics of maxillary sinus septa in the broader population, potentially limiting the generalizability of the findings. Additionally, being a single-center study conducted in the Madina region, the results may not be applicable to populations in different geographic areas or ethnic backgrounds, underscoring the need for caution in interpreting the study's outcomes. Future research should aim for more diverse and representative samples across various regions to provide a more comprehensive understanding of this anatomical variation.

## Conclusions

In the studied sample, 39.4% exhibited sinus septa, with variations in septum extension and location. No significant gender differences were observed. The detection and evaluation of these septa are crucial for radiologists and dental professionals, especially when planning surgical procedures like dental implant insertion or endoscopic sinus surgeries, to prevent complications. Prevalence rates for maxillary sinus septa differ across studies. The present study's findings align with some past research, while discrepancies exist with others regarding septa prevalence on the right versus the left side. Regardless of these variations, an in-depth understanding of maxillary sinus septa anatomy is imperative for dental professionals to ensure optimal surgical planning and patient outcomes.
